# Dopamine and sense of agency: Determinants in personality and substance use

**DOI:** 10.1371/journal.pone.0214069

**Published:** 2019-03-19

**Authors:** Anna Render, Petra Jansen

**Affiliations:** Faculty of Psychology, Pedagogic, & Sport Science, University of Regensburg, Bavaria, Germany; Western Sydney University, AUSTRALIA

## Abstract

Sense of agency refers to the feeling of control over one’s own actions. The strength of this sense varies inter-individually. This means that people differ in their perception concerning the intensity of their intentions and actions. The current study aims to determine the factors influencing this sense of agency on a personality level. Furthermore, it gives insight into the correlative relation between the strength of the sense of agency and substance use. The study involved 210 participants who were tested for the experiment (intentional binding paradigm for sense of agency, hand paradigm for intentionality bias, questionnaires FAD-Plus, NI-20, substance use). Significant determinants in personality were narcissism (vulnerable subtype) and substance use (consumption in general beyond cannabis, and particularly for the substances cannabis, ecstasy, and cocaine). Both personality types were associated with a weaker sense of agency compared to controls. For both results, alterations in the dopaminergic system need to be discussed. The present results confirm prior hypotheses that dopamine seems to play a crucial role in perception of agency. Possibly a higher accessibility of dopamine increases sense of agency (hyper-binding), whereas a lower accessibility of dopamine decreases sense of agency (hypo-binding). A second aim of the study was to see whether there is a connection between sense of agency and intentionality bias. The perception of intention in others differs widely; some people tend to see arbitrary or accidental actions as unintentional, and others quickly label actions as ‘intentional’ although the information is not distinct for a categorization. This cognitive error is called intentionality bias. Results could not confirm a relationship between the two constructs—one’s own intention and judging intention in others. This may be due to a lack of connection between the two constructs or to methodological aspects. Further directions and limitations are discussed.

## Introduction

Sense of agency, examined in this study, is defined by naming the cause of action [1]. It can be considered as a precondition for free will. People feel that they can create actions and are able to influence their surroundings. To measure the degree of consciousness of actions, an implicit paradigm called ‘intentional binding’ can be used to measure sense of agency. Intentional binding defines a time shift in perception between an intentional executed action and a following sensory event. If an action is intentional and feels controlled by the actor, a binding effect can be observed: the time between action and event is perceived as shorter than in reality; in other words there is a subjective compression of time [2]. The knowledge to be the cause of one’s own action is an elementary and constant root for the interaction with the world [3]. A possible explanation for alterations in sense of agency can be seen [4] in the ‘Comparator Model’ [5]. It postulates that the sensory consequences of actions can be predicted based on internal action-related information such as the efferent copy of a central motor command. In accordance with this model, sense of agency is defined as a retrospective inference relating to the causal structure of action and effect [6].

To identify determinants of sense of agency, we chose to examine the relation to certain personality factors and particularly substance use.

### Determinants of sense of agency: Current state of research

#### Sense of agency and personality

As to the relation of personality and sense of agency, the concepts of free will and narcissism have been investigated in previous studies. Free will and sense of agency both deliver information for the perception of action and events. They are similar constructs from different perspectives: free will as an explicit measure—the judgement of agency—and sense of agency as an implicit measure—the feeling of agency [7]. Narcissism was chosen because narcissists perceive themselves and their actions as particularly important and unique [8], a trait that has proved to influence sense of agency [9].

One study revealed that free-will beliefs are associated with conscious and unconscious processing of information involved in purposive behaviour and agency [10]. People with stronger beliefs in free will seem to concentrate on processing predictive signals of effects, thereby showing greater intentional binding than shown by individuals with weak beliefs in free will. This can be seen as a hint for a correspondence of stronger beliefs in free will and a stronger focus on the outcomes of actions. However, the number of studies rejecting a correlation between the implicit measure sense of agency and the explicit measures outweigh the ones finding a convergence [11–14].

Regarding narcissism, a first study examining differences in personality and intentional binding was published in 2015 [9]. People with medium and high scores of narcissism have a stronger sense of agency than do people with low narcissism scores. It is possible that narcissists experience themselves as highly effective agents and are more motivated and act more dominant than people with lower narcissism scores. However, it needs to be considered that none of the participants in that study reached a maximum score of narcissism, reducing the explanatory power of the results. Personality traits that correlate with low narcissism, such as low self-esteem, anxiety, and depression, have already been linked to a weaker sense of agency or weaker sense of control in previous studies [15,16]. Nonetheless, the study gives exploratory hints for personality being related to sense of agency [9]. It has been proposed to examine the differences in sense of agency for the two subtypes of narcissism—the vulnerable type and the grandiose type [17]—expecting a weaker sense of agency for the vulnerable type and a stronger sense of agency for the grandiose type [18].

#### Sense of agency and dopamine

The manner of relationship between the dopaminergic system and intentional control has not yet been understood. Dopamine is involved in responses to attention-inducing stimuli and reward-related stimuli [19]. It is part of the motivational reward system [20,21], but it is also involved in memory formation and motoric functions [22]. Additionally, dopamine regulates the prediction of errors in action results [19,19] and executive control [23]. The difference between the occurrence and the prediction of reward determines the reward response. This means that a temporal delay induces depression, and an unpredicted time of reward (perceived earlier than predicted) leads to activation [19]. Information linked to reward not only supports control of intentional acts [24,23] but also facilitates sense of agency. The activity of dopamine could increase the temporal binding of action and event (affecting the sense of agency: higher coherence and self-generation). Consequently, individuals with a low dopamine level will not profit from reward signals in their sense of agency [24].

Several studies have found hints for an involvement of the dopaminergic system in sense of agency. For example, one study used ketamine as a model for psychosis [25]. The aim of the experiment was to see whether ketamine creates the effect observed in schizophrenia in previous studies [26–30]. The participants were given low doses (100ng/mL plasma) of ketamine before performing the intentional binding task (measure for sense of agency). Ketamine increased intentional binding significantly compared to the placebo group. Moreover, ketamine significantly augmented the predictive influence on action binding. This effect is similar to the performance of patients with prodromal symptoms of schizophrenia [4].

Furthermore, psychosis-like experiences were seen to be correlated with an increased intentional binding; age was negatively related to intentional binding. Both results can be caused by alterations in the dopaminergic system [31]. There are findings for higher releases of dopamine, higher synaptic dopamine concentrations, and higher dopamine receptor occupation in people with experiences resembling psychosis [32–34], and dopamine levels in the brain decrease with age [35,36].

Another relevant study in view of the dopaminergic system includes Parkinsonism and sense of agency [37]. Dopaminergic medication is usually prescribed because Parkinson’s disease is accompanied by a loss of dopamine in the nigrostriatal pathway (38). While the motoric effects of dopaminergic drugs are relatively shaped, cognitive effects are less understood. The overdose theory claims that early stages of Parkinson’s disease lead to dopamine depletion in the dorsal striatum [38]. Consequently, dopaminergic medication has a positive effect on cognitive functions. At the same time, there is little dopamine depletion in the ventral striatum, so that dopaminergic drugs interfere in this region through a dopaminergic overdose. In a review [39] this was illustrated as an inverted U-shaped relationship between dopamine levels and performance. The patient’s individual genotype has a major influence defining the relative baseline position on the curve and the interaction with the dopaminergic medication and thereby explains (different) interpersonal reactions and performances. The individual genotype (for genetic polymorphisms) determines the dopaminergic metabolism and availability of dopamine. This study [37] first tested patients with Parkinson’s off medication, then on medication (L-Dopa, short-acting dopamine agonist) and compared these results with a healthy control group. It seems that higher accessibility of dopamine increases the intentional binding effect (sense of agency). The highest binding effect was found in patients on medication, whereas patients off medication and the control group did not differ in their sense of agency. Corresponding to this, Parkinson’s alone is not associated with alterations in sense of agency. Dopaminergic medication boosts the action-binding effect. The significant effect lies in the linkage of action and effect binding and not action binding or effect binding individually. This means that the linkage might be experienced differently, but not the tone or action itself.

The studies on L-Dopa medication in Parkinson’s and on ketamine indicate the relevance of the dopaminergic system to sense of agency. Dopamine increases the spectrum for the binding strength of two events. It is possible that the probability to include external stimuli is increased by a high dopamine concentration and decreased by low dopamine activity [31]. This raises the question of whether and how a usage of other substances influences sense of agency, particularly with respect to long-term changes, not only in acute intoxication. For clarification, further investigation of substance use and possible links to changes in sense of agency is needed. Less dopamine accessibility seems to be linked to a weaker sense of agency. It has to be investigated whether having an affinity to use drugs that change receptor mechanisms in the dopaminergic system is related to a weaker sense of agency in the long run.

#### Sense of agency and substances

Already 20 years ago researchers suspected that chronic use of drugs could induce changes in the neurotransmitter systems, particularly the dopamine system [40]. Hence, it is important to investigate whether alterations in the dopaminergic system, caused by drug use, lead to differences in sense of agency.

The substances in focus are cannabis, MDMA (e.g. ecstasy), cocaine, amphetamine (e.g. speed), and psychedelics (ketamine, LSD, and mushrooms). Therefore, we concentrate rather on long-term effects of substance use because none of the subjects will be tested for acute intoxication of drugs.

Use of cannabis, contrary to use of other substances, is not associated with striatal dopamine alterations. However, observable alterations (lower dopamine release in the associative striatum) have been found in users who started early or reported a long duration of usage. Consequently, it is difficult to distinguish the effects of chronicity versus onset [41]. Ecstasy (MDMA, MDEA, MDA), an activating and hallucinogenic substance, directly affects the neurotransmitter metabolism. In animal trials, an increase of serotonin level in the synaptic cleft could be observed, which probably causes changes in the dopamine systems. Release of serotonin could also be responsible for a higher dopamine release [42]. In apes, high doses change the serotonin system irreversibly, mostly affecting parts of the brain responsible for memory processes and development of anxiety [43]. Several other studies have confirmed that the level of dopamine D2 receptor availability is lower than normal in drug-addicted subjects (alcoholics, cocaine abusers, crystal abusers, heroin abusers) [44]. Cocaine use results in long-term reduction in the hypothalamic and frontal cortex dopamine metabolism [45]. Even a single cocaine exposure in mice leads to alterations in the dopamine metabolism; 10 days after administration, receptors of dopamine cells are blocked [46]. Studies in rats suggest that amphetamines produce a long-term striatal dopamine depletion by destroying striatal dopamine nerve fibres [47], and even a single exposure to amphetamine seems to be sufficient to induce long-term behavioural, neurochemical, and neuroendocrine sensitization [48]. In regard to psychedelics, ketamine is a NMDA receptor antagonist that influences the dopaminergic and glutamatergic functions. Prediction error is understood to be a mismatch between the agent’s expectation (from his or her environment) and real events, regulated by dopaminergic and glutamatergic mechanisms [49]. In animal studies, chronic dopamine depletion, induced by chronic ketamine use, produces selective up-regulation of D1 receptor availability (in the dorsolateral prefrontal cortex) [50]. The increased availability can be due to augmented receptor density or affinity. The D1 receptor up-regulation might be caused by a drug-induced deficit in prefrontal dopamine function. This up-regulation of D1 receptors could be an attempt to compensate for the deficit. Repeated use of ketamine produces an alteration of the prefrontal dopaminergic transmission. Parallels to the effects of chronic ketamine use can be seen in schizophrenic patients [50]. Chronic ketamine use produces a deficit of prefrontal dopamine function. Although a receptor up-regulation seems to compensate, still less dopamine might be available. LSD is known to take effect in two phases, with the later temporal phase mediated by D2 dopamine receptor stimulation. [51]. Even a single dose of LSD increases the expression of a small set of genes in the mammalian brain that are involved in a wide array of cellular functions, which reflects the beginnings of long-term neuro-adaptive processes [52].

It is likely that people consuming drugs have developed different mechanisms in the dopaminergic system, resulting in a weaker sense of agency (if less dopamine is available, in abstinence) or a stronger sense of agency (if more dopamine is available, in acute consumption of drugs).

### Sense of agency and intentionality bias

In addition to the determinants of sense of agency in personality and substance use, we aim to investigate how the sense of agency relates to perception of intention in others. Until now, no research has combined perception of one’s own intention and evaluating the intention of others. Do we expect others to have the same level of control as we feel to have ourselves? Alternatively, do we measure their actions and intentions with different standards from our own actions?

Concerning accidental or arbitrary actions of others, some people show a bias towards interpreting actions as intentional. If an action was performed unintentionally, but the information about the intention of action is missing, we still tend to label it as intentional. This construct is called intentionality bias [53].

Rosset and Rottmann [54] developed a model with two routes of explaining the processing for deductive reasoning in consideration of the intention of others. The first rapid and automatic response to behaviour activates the intentionality bias. At first, all actions are categorised as intentional. In a second process, a deliberate, more accurate analysis can overwrite this bias in case of evidence for unintentional behaviour. According to this theory, classifying behaviour is determined not only by the skill to recognize hints for intention but mainly through the skill to identify errors of interpretation and the ability to overwrite these errors [54].

Until now, nothing has been known about the relationship of the two constructs. Is there a connection between the degree of feeling in control of one’s own actions (sense of agency) and the interpretation of intention (attribution of control) in other people’s actions (intentionality bias)? And if so, do people with a higher sense of agency (higher feeling of control for their actions) suspect more actions as intentional by others as well?

### Goals and hypotheses

The first goal of this study is to find determinants for sense of agency in personality and substance use.

1. In terms of personality factors, evidence exists for a stronger sense of agency in narcissists [9]. We extend the current state of research by differentiating between the vulnerable and grandiose type of narcissism [17], expecting higher sense of agency in people with grandiose narcissism and lower sense of agency in people with vulnerable narcissism [18]. Methodically, implicit (intentional binding) and explicit (FAD-Plus) measurements of sense of agency do not correlate with each other [13,12,11].
2. Recent experiences have manifested that dopaminergic drugs boost sense of agency under intoxication [37,25]. Substance use might influence the sensitivity of receptors and/or transporters of the dopaminergic system, affecting sense of agency. A drug-induced deficit in the dopaminergic function [50] could cause alterations in sense of agency. Lower availability of dopamine is associated with a weaker sense of agency [24,31]. We hypothesize a negative link between sense of agency and consuming the substances under investigation. Using drugs might (in the long term) go along with alteration in the dopaminergic system (lower dopamine levels), attributing for a weaker sense of agency.

The second goal of the current study is to determine whether the perception of intention of one’s own action (sense of agency) and the evaluation of intention in the actions of others (intentionality bias) are connected. Consequently, the third (non-directional) research question is whether sense of agency and intentionality bias correlate with each other. If the two constructs are linked, it would be interesting to learn the following: Is the connection positive, meaning that the more we perceive our own action as voluntary, the more we presume intention in others? Or is the link negative, meaning that the more we act voluntarily, the less we expect others to act voluntarily?

## Materials and methods

### Power analysis

To estimate the sample size necessary to find effects, power analyses were run. Previous research in narcissism has found overall binding values for high narcissism scores *M* = -157.40 (*SD* = 51.08) and low narcissism scores *M* = -100.20 (*SD* = 45.94) [9]. Power analysis, one tailed for independent *t*-tests with *α* = .05, *β* = .95, and Cohen’s *d* = 1.177 calculated a necessary sample size of *N* = 34.

An experiment using ketamine as a model for psychosis reported overall binding values of placebo *M* = 45 (*SD* = 69), ketamine administration M = 72 (*SD* = 70) [25]. Power analysis, one tailed for independent *t*-tests with *α* = .05, *β* = .95, and Cohen’s *d* = .388 calculated a necessary sample size of *N* = 145. Although the power analysis for narcissism calculated that only a small sample size is required, more participants were recruited with regard to effects for the different substances.

### Sample

We tested 210 participants for the experiment. Participants were informed about the purpose of the study and gave their written consent prior to participation. The experiment was conducted according to the ethical guidelines of the Helsinki Declaration. Data were analysed anonymously. Participants gave information about their age, ranging from 17 to 34 years, *M* = 23.33, *SD* = 3.52, and sex; 84 (40.0%) participants were male, 126 (60.0%) female. IQ measured by the Trail Making Test was *M* = 118.35, *SD* = 15.97, ranging from 88 to 145, to ensure that both groups (drug users and controls) were demographically similar. Ten participants were not students; the other participants were students who studied psychology (30), sport sciences (99), arts and humanities (36), criminology (4), law (5), natural science (8), and other disciplines (18).

We did not seek approval from a research ethics board because it was not required for this study in accordance with conditions outlined in guidelines from the German Research Society (DFG, Deutsche Forschungsgesellschaft): Research bearing no additional risk beyond daily activities does not require such approval. We communicated all considerations necessary to assess the ethical legitimacy of the study. We thus ensure that our research approach is in line with national and international human research ethics policies.

### Apparatus and stimuli

#### Intentional binding

To assess intentional binding (sense of agency), the method of Haggard et al. [2] was applied as a guiding procedure. The experimental paradigm was generated by the description of Aarts and van den Bos [10]. The experiment was programmed by modifying code from new HTML5 Application Programming Interfaces (APIs) that maximize accuracy and timing precision. These include the following features: 1) CSS animations for presenting visual stimuli, 2) web audio API for presenting auditory stimuli, and 3) DOM event timestamps for logging user interaction [55]. The participants watched an analogue clock, marked with numbers in intervals of 5 (0, 5, 10, 15, 20, 25, 55). The duration of one clock rotation was 2560ms. In each trial, the clock rotated two times; the event occurred in all trials in the second lap. There were four different conditions: two baselines and two agency blocks. The order of conditions was counterbalanced between subjects.

1. *Baseline action*: The participants watched the analogue clock and pressed the key (space) whenever they wanted to in the second lap. Afterwards they reported the time of pressing the key (on the analogue clock).
2. *Baseline outcome*: The participants watched the analogue clock and heard a tone at a random time. Afterwards they reported the time of hearing the tone (on the analogue clock).
3. *Agency action*: The participants watched the analogue clock and pressed the key (space) whenever they wanted to in the second lap. A tone followed with a delay of 250ms. Afterwards the participants reported the time of pressing the key (on the analogue clock).
4. *Agency outcome*: This condition was identical to agency action, but this time the participants reported the time of hearing the tone (on the analogue clock).

In the three conditions when the participants had to press a key (conditions 1, 3, and 4), they were asked to let the clock rotate once before pressing the key and not to press the key at a certain time (always at the same time or only at the interval marks of 5). In addition, the instruction indicated that they should be as precise as possible (in intervals of 1). Each block contained 18 trials, according to Moore et al. [37].

#### Intentionality bias

**Fig 1:**
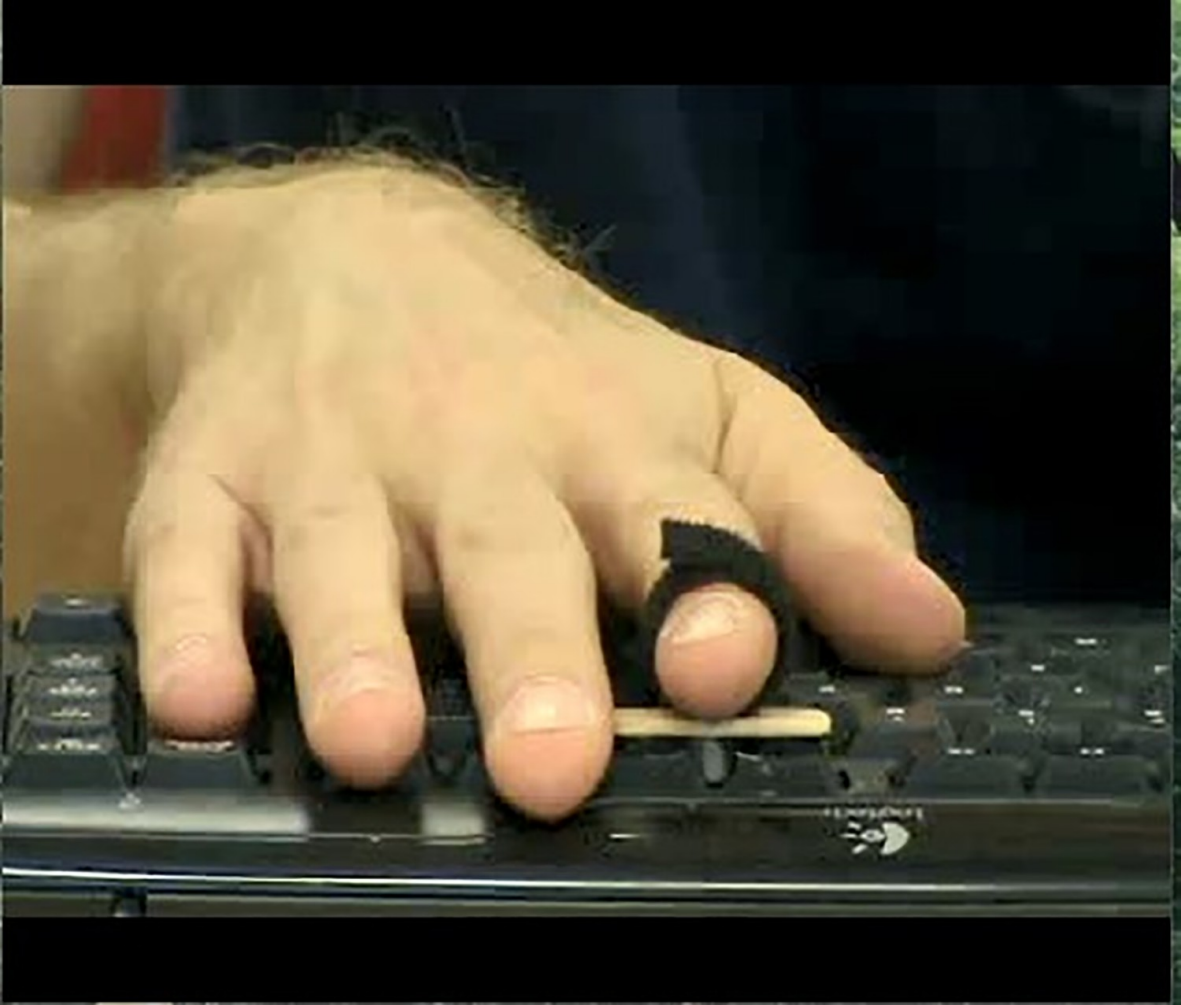
Intentionality bias paradigm. Screenshot of video by Moore and Pope [56] for the intentionality bias paradigm.

To guarantee that language (grammar, choice of words) did not influence the task, a non-verbal task, developed by Moore and Pope [56], was used in the experiment. The participants watched videos in which a hand whose finger was linked to a keyboard could be seen (see Fig 1). In the video, the finger was pulled down toward the keyboard. The participants were asked to decide whether the finger actively pressed the key (intentional) or was pulled down by the keyboard (not intentional). In all videos, the finger was pulled down by the keyboard, representing a non-intentional condition. The videos with four different onset times until the finger was moved were presented 26 times. Two of them were exercise trials and not included in the analysis. The 24 trials were taken together (0 = not intentional, 1 = intentional), and this score in percent evaluated the strength of the intentionality bias for each person.

#### Trail Making Test

The Trail Making Test (ZVT; [57]) measures cognitive processing speed. The test consists of four pages, where the numbers 1 to 90 are arranged in a scrambled order in a matrix of 9 rows and 10 columns. The participants had to connect the numbers as quickly and correctly as possible in ascending order, measuring the total time for each page. The test duration was about 5 minutes. The evaluation revealed ZVT scores, which were transferred to corresponding IQ values. The correlation (e.g. Raven-SPM, CFT-30) ranged between r = .60 to .80. The test–retest reliability as well as the internal consistency of the ZVT was about .90 to .95 [58].

#### Questionnaires

**Demographics**. Participants reported sex, age, studies, and profession.

**Narcissism Inventory**. Narcissism is measured by the Short Version of the Narcissism Inventory (NI-20) of Daig et al. [59]. The basic concept is the narcissism inventory (NI-90) of Schoeneich et al. [60]. The short version is composed of four factors: 1) threatened self, with eight subscales (helpless self, loss of control over affects and impulses, de-realization/depersonalization, basic potential of hope, worthless self, negative bodily self, social isolation, and withdrawal into feelings of harmony); 2) classic narcissistic self, including four subscales (self-grandiosity, longing for an idealized self-object, greed for praise and reassurance, and narcissistic furore); 3) idealistic self, with four subscales (self-reliance ideal, object devaluation, idealizing values, and symbiotic self-protection); and 4) hypochondriac self, with the subscales of hypochondriac expression of fear and narcissistic gain from illness. The Cronbach’s alpha was above .7. The factor structure was confirmed by an exploratory and confirmatory factor analysis. Moreover, it was normed and compared by samples, *N* = 2262 and *N* = 2265 [59]. Threatened self represents the vulnerable type and classic narcissistic self the grandiose type; only these two scales will be used for analysis.

**FAD-Plus**. The FAD-Plus covers four scales: free will, scientific determinism, fatalistic determinism, and unpredictability, with 27 items in total. It was normed by *N* = 257, *N* = 177, and *N* = 188 participants. Concerning quality criteria, the authors mention reliability (Cronbach’s alpha .69 to .82 for the subscales). The four factorial structure was conformed in a confirmatory analysis [61].

**Substances**. The items to gather information about the substance use of the participants were self-generated from the AUDIT [62] questionnaire of the addiction research network Baden-Württemberg UKL Freiburg, classifying the use of alcohol. There were two categories: ever consumption and prior-year average consumption of alcohol, nicotine, prescription pharmaceuticals (painkiller, tranquilizer, medication for physiological diseases), and illegal drugs (cannabis, amphetamine, ecstasy, LSD, mushrooms, ketamine, cocaine). In addition, the amount, frequency, and years of use were registered for each substance.

### Procedure and experimental design

The study took part at the University of Regensburg, and the duration was roughly 50 minutes. Sessions started with the ZVT [57], followed by the computer experiment of intentionality bias [56] and intentional binding [2,10]. The second part included the online questionnaires generated with Sosci-Survey. The ZVT and two computer-based experiments took about 30 minutes, and the questionnaires 20 minutes.

### Statistical analysis

To calculate intentional binding in each trial, perceived time was subtracted from actual time to determine the perception error. First, action binding and outcome binding were calculated individually. Action binding is composed of baseline action minus agency action, and outcome binding of baseline outcome minus agency outcome (to generate the difference from baseline to the operant conditions). Outcome binding was then subtracted from action binding to calculate overall binding [25,37]. Medians instead of means were used to eliminate outliers [63].

The Pearson correlation coefficient was calculated to examine relationships between the two constructs (sense of agency and intentionality bias), the link between sense of agency and personality factors, symptoms of disorder, and substance use. One-tailed t-tests were conducted for the highest versus lowest percentiles in narcissism types and different groups of substance users versus the control group.

The alpha error accumulation for the three questionnaires was calculated by using the formula 1 –(1–0.03)^3^. This means that the alpha error was 8.73% instead of 5%; therefore, the level of significance needed to be adjusted. The significance level was corrected according to Bonferroni, and the *p*-value was set to *p* = .017 to reach significance.

## Results

### Determinants of sense of agency

To understand the nomological network of the sense of agency and the examined variables, Table 1 shows all correlation coefficients to sense of agency, and Table 2 focuses on frequency of use of different substances. Only the correlation for ketamine reached significance.

**Table 1:** Pearson Correlation Coefficients for determinants and sense of agency.

Variable	*r*	*p*
**Age (one-tailed)**	-.103	.073
**IQ (two-tailed)**	-.011	.878
**Personality (one-tailed)**		
Threatened Self (vulnerable)	-.146	.020
Hypochondriac Self	-.007	.461
Classic Narcissistic Self (grandiose)	-.076	.142
Idealistic Self	.029	.342
Narcissism (overall)	-.078	.135
**Beliefs (two-tailed)**		
Free will	-.093	.191
Scientific Determinism	-.007	.918
Fatalistic Determinism	.161	.022
Unpredictability	.056	.432
**Intentionality Bias (two-tailed)**	-.101	.154

**Significance**: *p* ≤ .017

**Table 2:** Pearson Correlation Coefficients (one-tailed) for frequency of use and sense of agency.

Frequency of Use	*r*	*p*
**All participants**		
Alcohol	-.095	.140
Smoking	.108	.079
Cannabis	-.056	.222
**Drugs (users only)**		
Tranquilizers	.143	.106
Psychotropics	.029	.400
Cannabis	.185	.062
Amphetamine	.168	.071
Ecstasy	.196	.043
LSD	.220	.027
Mushrooms	.230	.022
Ketamine	.250	**.014**
Cocaine	.136	.118

**Significance**: *p* ≤ .017, Users only = consumption beyond cannabis *N* = 78

#### Sense of agency and demographics

The mean intentional binding was *M* = 84.70 (*SD* = 136.41). Negative values signify that the event is perceived later than it really was, and positive values indicate that the event is perceived earlier than it really occurred. The mean shift in the baseline action was *M* = -50.79 (*SD* = 95.62), mean shift in the agency action condition was *M* = -90.91 (*SD* = 117.55), mean shift in the baseline outcome condition was *M* = -59.40 (*SD* = 55.33), and mean shift in agency outcome was *M* = -14.18 (*SD* = 130.24). Action binding (baseline action minus agency action) was *M* = 41.52 (*SD* 77.77), and outcome binding (baseline outcome minus agency outcome) was *M* = -45.17 (*SD* = 122.01). No difference (in overall binding) could be observed for sex (male *M* = 81.67, *SD* = 142.50, female *M* = 86.73, *SD* = 132.74, *t*(200) = -.258 *p* = 797), age (19–34, weaker sense of agency than in older people), or IQ. Due to technical errors, data were missing from eight participants in intentional binding (drug users = 3, controls = 5 missing).

#### Sense of agency and personality

Contrary to results previously reported in the literature, no correlation to general narcissism (*r* = -.078 *p* = .269) was found. Splitting the sample into the first and fourth percentiles of the subscales threatened self (vulnerable type) and classic narcissistic self (grandiose type), the following results were produced. The vulnerable type had a weaker shift in perception of the key press if the tone followed (agency action trials), and consequently a weaker sense of agency (total binding), than people with low scores of vulnerability (see Table 3). Independent t-tests showed differences (*t*(2470) = -7.529 *p* < .001) from the means of threatened self in this study (*N* = 209, *M* = 1.920, *SD* = .635) compared to the means in the norms (*N* = 2262, *M* = 2.620, *SD* = 1.330), and threatened self was significantly smaller in the current study in comparison to mean norms (patients from Charité Clinic, Berlin).

**Table 3:** Sense of agency and threatened self.

Subscale	*M*_high_	*SD*	*M*_*low*_	*SD*	*t*-test	*p*
**Baseline action**	-47.00	91.50	-54.94	95.08	-.391	.349
**Agency action**	-68.48	88.93	-127.77	132.12	-2.689	**.004**
**Baseline outcome**	-53.85	53.58	-65.74	49.81	-1.186	.119
**Agency outcome**	-26.29	110.11	-14.20	152.75	-.469	.320
**Total binding**	53.22	122.32	118.36	172.88	2.208	**.015**

One-tailed *t*-tests for intentional binding conditions, first (high, *N* = 50) versus fourth (low, *N* = 55) percentiles in the narcissism subscale threatened self. **Significance** *p* ≤ .017.

The grandiose type discriminated neither in binding nor in the sub-conditions, as presented in Table 4. Means in this study and means from the norms did not differ from each other (this study *N* = 209, *M* = 2.375, *SD* = .645, norms *N* = 2262, *M* = 2.520, *SD* = 1.190, *t*(2470) = -1.737 *p* = .083).

**Table 4:** Sense of agency and classic narcissistic self.

Subscale	*M*_high_	*SD*	*M*_*low*_	*SD*	*t*-test	*p*
**Baseline action**	-59.35	102.20	-61.26	94.64	-.097	.462
**Agency action**	-82.10	108.20	-102.44	119.57	-.883	.190
**Baseline outcome**	-65.50	65.68	-60.20	58.04	.430	.334
**Agency outcome**	-19.62	118.50	-4.00	142.45	.589	.279
**Total binding**	68.63	106.22	91.46	147.00	.867	.194

One-tailed *t*-tests for intentional binding conditions, first (high, *N* = 44) versus fourth (low, *N* = 56) percentiles in the narcissism subscale scores for classic narcissistic self. **Significance** *p* ≤ .017.

All explicit measures of beliefs measured by the FAD-Plus, in particular the subscale free will and sense of agency, as an implicit measure, were independent (*r* = .09 *p* = .21).

#### Sense of agency and substances

**Demographics of Users and Controls**. Comparing both groups, drug users were on average three years older than controls (users’ age *M* = 25.01, *SD* = 3.71, controls *M* = 22.27, *SD* = 2.94, *t*(208) = -5.931 *p* < .001), and IQ was lower in the group of drug users (controls *M* = 120.72, *SD* = 15.61, users *M* = 114.51, *SD* = 15.87, *t*(197) = 2.712 *p* = .007). There were different gender ratios, the control group had more female members (*N* = 129, 40 male, 89 female), the experimental group more male members (*N* = 81, 44 male, 37 female). The differences in demographics are not expected to explain alterations in sense of agency (no differences for demographics for all participants in intentional binding, see Sense of Agency and Demographics).

Table 5 lists how many participants consumed each substance (ever consumed).

**Table 5:** Consumed substances (Ever).

	Cannabis	Ecstasy	Amphetamine	Mushrooms	LSD	Cocaine	Ketamine	> Cannabis
*yes*	131	63	57	44	43	49	34	81
*No*	78	146	152	165	166	160	175	129

Absolute number of users.

In regard to substance use (ever), there were several differences in binding (see Table 6). Independent t-tests showed significant differences in sense of agency for drug users who had consumed cannabis, ecstasy, or cocaine and new variable computed by summarizing all users who had consumed other drugs besides cannabis (at least one additional substance) compared to the control group: Consumers had a significantly weaker sense of agency than did controls.

**Table 6:** Differences in sense of agency (overall binding) between drug users and controls.

Substance	*M*_users_	*SD*	*M*_control_	*SD*	*t*-test	*p*
**Cannabis**	67.51	136.31	111.29	133.13	2.227	**.014**
**Ecstasy**	51.87	139.51	97.77	133.28	2.203	**.015**
**Amphetamine**	60.80	141.07	92.84	134.12	1.489	.069
**Mushrooms**	67.49	132.90	88.58	137.47	.898	.185
**LSD**	66.29	147.33	88.63	133.63	.935	.176
**Cocaine**	43.74	124.84	96.38	137.85	2.340	**.010**
**Ketamine**	76.42	138.23	85.63	136.45	.359	.360
**> Cannabis**	55.37	134.50	103.15	134.26	2.454	**.008**

One-tailed t-tests for sense of agency in users versus controls for each substance. **Significance** *p* ≤ .017. Drug users = consumption beyond cannabis.

**Fig 2:**
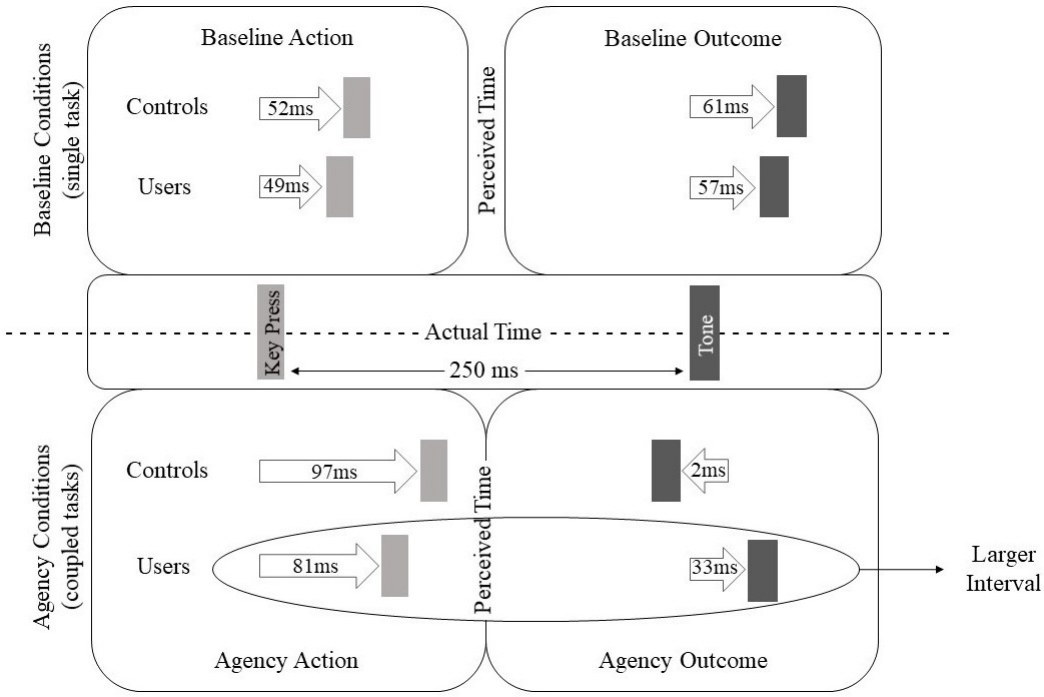
Intentional binding of drug users versus controls. Illustrated are shifts in perception (difference = actual time minus perceived time) for baseline action (1), baseline outcome (2), agency action (3), and agency outcome (4). Key press = action, tone = event. Drug users = consumption beyond cannabis.

Fig 2 illustrates the alterations in perception. One can conclude that drug users have greater intervals of binding between action (key press) and event (tone), due to later perception of the time of tone.

### Sense of agency and intentionality bias

The analysis rejects any association between sense of agency and intentionality bias. Apparently there is no connection between how people perceive the control of their own actions and how they regard the control and intention in the actions of others (*r* = -.101 *p* = .154).

## Discussion

### Determinants of sense of agency

The current study found significant determinants for sense of agency in personality, in the vulnerable subtype of narcissism, and for several substances in drug users (cannabis, ecstasy, cocaine, substances beyond cannabis). Both groups showed a decrease in intentional binding; they seemed to have a weaker link between action and consequence. A relation between the explicit (FAD-Plus) and implicit measure (intentional binding) of free will could not be found, nor was there a connection between intentionality bias and sense of agency (intentional binding).

#### Sense of agency and personality

Previously, in narcissists, a stronger sense of agency has been found [9]. In this study, we distinguished between different forms of narcissism, because there has been evidence that narcissism occurs in two subtypes: the vulnerable and the grandiose type [64]. Dimaggio and Lysaker [18] supposed that the two types of narcissists also differ in their sense of agency. The vulnerable subtype could be more closely linked to reduced agency (e.g. when being rejected in a romantic relationship, which impairs an already unstable self-esteem [65]), while the grandiose type experiences hyper-agency. This is a possible explanation for our results. It can be seen in the factor of the threatened self, which contains—among others—the aspects of helpless self, worthless self, negative bodily self, and social isolation. These facets embody the vulnerability–sensitivity type [59], the covert form of narcissism. Our results confirm a lower sense of agency in the highest percentile compared to the lowest percentile. Grandiosity–exhibitionism is represented by the classic narcissistic self [59]. The hypothesis for a stronger sense of agency in people scoring high on this subscale cannot be confirmed. Results from previous studies have shown negative correlations between social desirability and narcissism questionnaires, which may explain the lack of results for grandiose narcissism (caused by covering narcissistic tendencies/personality traits [66]).

A recently published study differentiated also between the two subtypes of narcissism, grandiose type and vulnerable type. These subtypes are theoretically (biologically) based on the behavioural approach (BAS, sensitivity to reward) and behavioural avoidance (BIS, sensitivity to punishment) systems of personality (Gray) [67]. The study links the subtypes of narcissism to possible alterations in the dopaminergic systems [68]. High BAS and low BIS predicted grandiose narcissism; moderate BAS and high BIS predicted vulnerable narcissism. A strong imbalance between the motivational systems BIS and BAS can be predicted by changes in the dopaminergic system [69]. It is possible that the underlying motivational concepts explain the differences found in the vulnerable narcissism subtype. This is a first hint for a mediating role of dopamine in narcissism and sense of agency.

#### Sense of agency and substances

Being experienced with multiple drug use (consumption besides cannabis) in general, and particularly with the consumption of cannabis, ecstasy, or cocaine was significantly related to a weaker sense of agency in our experimental subjects. In the baseline conditions, the values of perception were similar, negating a prolonged perception in general for drug users. However, changes occur if actions and consequences are presented in the same trial. Usually people tend to compress the time between action and event. In drug users, in our study, the tone was perceived later, which extended the interval between action and consequence.

The number of participants in this study limits the generalization of results to specific substances. In addition, the majority of the examined participants reported poly-drug use and variations in amount, frequency, and years of use, which limited the examination for single substances. At the same time, the study included participants who reported only a single drug consumption. The sample was heterogeneous; therefore, use of drugs seemed to produce a robust effect in the binding mechanism. Effects of single substances remain too difficult to interpret. However, their connecting feature is the possible alteration of the dopaminergic system.

### Sense of agency and intentionality bias

In the current data, no correlation could be found between the constructs sense of agency and intentionality bias. The experimental paradigm was conducted very simply to control potential influencing factors. Engbert et al. [70] conducted three experiments, drawing the conclusion that sense of agency is relational, generalizable, requires efferent motor commands, and is private rather than socially shared. The last aspect includes the fact that people do not simulate the agency of others. This could be one explanation for the independence of sense of agency (in oneself) and intentionality bias (in others) in this study. Considering that this is the first study, to our knowledge, to investigate the relationship between the two constructs, it is possible that this lack of connection is due to methodological issues and that a connection could be found in a different operationalization of measurements.

“Too often, we judge other groups by their worst examples—while judging ourselves by our best intentions” (Bush, 2016). We judge ourselves by our intentions; we judge others by their actions. This citation emphasizes that judging our own intentions may differ widely, but systematically, from understanding intention and actions in others. In contrast to the highly controlled laboratory experiment, in the outside world various potential aspects influence the judgement, for example situational factors, personality and mood factors, or the relationship of agents. Again, operationalization might be a highly contributing factor for the present results.

### Limitations and future research

In this study we investigated alterations in sense of agency in students, with an emphasis on illegal drug users. A sample of patients suffering from addictions, in a quasi-experimental design, could have delivered more distinct results. Alternatively, it would be interesting to examine people who consumed only a single substance, although recruiting such a homogenous sample might be an obstacle. Nevertheless, the results indicate an involvement of dopamine for sense of agency, even on a level that does not rise to a clinical one. Possibly the determination of the dopamine level could reveal whether higher accessibility of dopamine leads to hyper-binding and lower accessibility to hypo-binding. However, dopamine levels vary significantly, even intra-individually, so that many confounding factors would need to be controlled. Another possibility could be to determine gene types and genetic polymorphism involved in the dopaminergic metabolism (e.g. a transporter gene). For example, there are two variants of the COMT enzyme (COMT-Met versus COMT-Val), that increase or decrease the dopamine level [71]. It could be useful to determine the enzyme type of each person and connect it to the strength of sense of agency.

In regard to narcissism, it would be interesting to take a closer look at the underlying motivational systems BIS and BAS. A clinical sample could produce stronger effects. In this study, the participants did not report high narcissistic traits. Subsequent studies could focus on a comparison between healthy individuals and the two subtypes of narcissism. Additionally, other mental diseases like attention deficit hyperactivity disorder [72] or eating disorders (anorexia nervosa [73]), which are discussed as having alterations in the dopamine metabolism, or emotional states releasing dopamine (e.g. arousal, see [74]), could be investigated as well.

Self-report questionnaires bear the risk of misreport and social desirability, especially in narcissism questionnaires. A second measure, more independent and objective than self-reports, could offer more elucidation. Sense of agency was measured with a validated paradigm [2]. Since then, the experiment has been adapted by several research teams to get more detailed information about the subdivision of predictive and postdictive parts of sense of agency. However, in this study, the standard deviation was remarkably high, although the code written by Pablo Garaizar [55] is very accurate. This could indicate that sense of agency differs widely among the participants. It would be interesting to observe the stability of intentional binding from a retest reliability perspective. Yet to our knowledge, no study has been conducted that has investigated intra-individual differences in intentional binding over a longer period, at different times a day, or under certain conditions, e.g. emotional states or stress.

## Supporting information

S1 Table Pearson Correlation Coefficients for determinants and sense of agency. **Significance:** *p* ≤ .017.
(PDF)

S2 Table Pearson Correlation Coefficients (one-tailed) for frequency of use and sense of agency. **Significance**: *p* ≤ .017, Users only = consumption beyond cannabis *N* = 78.
(PDF)

S3 Table Sense of agency and threatened self. One-tailed *t*-tests for intentional binding conditions, first (high, *N* = 50) versus fourth (low, *N* = 55) percentiles in the narcissism subscale threatened self. **Significance** *p* ≤ .017.
(PDF)

S4 Table Sense of agency and classic narcissistic self. One-tailed *t*-tests for intentional binding conditions, first (high, N = 44) versus fourth (low, N = 56) percentiles in the narcissism subscale scores for classic narcissistic self. **Significance** *p* ≤ .017.
(PDF)

S5 Table Consumed substances (Ever). Absolute number of users.
(PDF)

S6 Table Differences in sense of agency (overall binding) between drug users and controls. One-tailed *t*-tests for sense of agency in users versus controls for each substance. **Significance** *p* ≤ .017. Drug users = consumption beyond cannabis.
(PDF)

S1 Data file (SAV)
