# Supplementary material for: Dopamine and sense of agency: Determinants in personality and substance use
Source: PLoS One. 2019 Mar 19;14(3):e0214069. doi: 10.1371/journal.pone.0214069 (PMC6424396; doi:10.1371/journal.pone.0214069)
Supplement: S1 Table — Significance: p ≤ .017. (PDF) [file pone.0214069.s001.pdf]

Table 1. Pearson Correlation Coefficients for Determinants and Sense of Agency.

**Variable**	***r***	***p***
**Age (one-tailed)**	-.103	.073
**IQ (two-tailed)**	-.011	.878
**Personality (one-tailed)**		
Threatened Self (vulnerable)	-.146	.020
Hipochondriac Self	-.007	.461
Classic Narcissistic Self (grandiose)	-.076	.142
Idealistic Self	.029	.342
Narcissism (overall)	-.078	.135
**Beliefs (two-tailed)**		
Free will	-.093	.191
Scientific Determinism	-.007	.918
Fatalistic Determinism	.161	.022
Unpredictability	.056	.432
**Intentionality Bias (two-tailed)**	-.101	.154

Significance: $p \leq .017$.
