# Supplementary material for: Dopamine and sense of agency: Determinants in personality and substance use
Source: PLoS One. 2019 Mar 19;14(3):e0214069. doi: 10.1371/journal.pone.0214069 (PMC6424396; doi:10.1371/journal.pone.0214069)
Supplement: S2 Table — Significance: p ≤ .017, Users only = consumption beyond cannabis N = 78. (PDF) [file pone.0214069.s002.pdf]

Table 2. Pearson Correlation Coefficients (one-tailed) for Frequency of Use and Sense of Agency.

**Frequency of Use**	***r***	***p***
**All participants**		
Alcohol	-.095	.140
Smoking	.108	.079
Cannabis	-.056	.222
**Drugs (users only)**		
Tranquilizers	.143	.106
Psychotropics	.029	.400
Cannabis	.185	.062
Amphetamine	.168	.071
Ecstasy	.196	.043
LSD	.220	.027
Mushrooms	.230	.022
Ketamine	.250	**.014**
Cocaine	.136	.118

Significance: $p \leq .017$, Users only = consumption beyond cannabis $N = 78$
