# Supplementary material for: Dopamine and sense of agency: Determinants in personality and substance use
Source: PLoS One. 2019 Mar 19;14(3):e0214069. doi: 10.1371/journal.pone.0214069 (PMC6424396; doi:10.1371/journal.pone.0214069)
Supplement: S3 Table — One-tailed t-tests for intentional binding conditions, first (high, N = 50) versus fourth (low, N = 55) percentiles in the narcissism subscale threatened self. Significance p ≤ .017. (PDF) [file pone.0214069.s003.pdf]

Table 3. Sense of Agency and Threatened Self.

**Subscale**	M_{high}	*SD*	M_{low}	*SD*	***t*-test**	***p***
**Baseline action**	-47.00	91.50	-54.94	95.08	-.391	.349
**Agency action**	-68.48	88.93	-127.77	132.12	-2.689	**.004**
**Baseline outcome**	-53.85	53.58	-65.74	49.81	-1.186	.119
**Agency outcome**	-26.29	110.11	-14.20	152.75	-.469	.320
**Total binding**	53.22	122.32	118.36	172.88	2.208	**.015**

One-tailed *t*-tests for intentional binding conditions, first (high, $N = 50$) versus fourth (low, $N = 55$) percentiles in the narcissism subscale threatened self. **Significance** $p \leq .017$.
