# Supplementary material for: Dopamine and sense of agency: Determinants in personality and substance use
Source: PLoS One. 2019 Mar 19;14(3):e0214069. doi: 10.1371/journal.pone.0214069 (PMC6424396; doi:10.1371/journal.pone.0214069)
Supplement: S4 Table — One-tailed t-tests for intentional binding conditions, first (high, N = 44) versus fourth (low, N = 56) percentiles in the narcissism subscale scores for classic narcissistic self. Significance p ≤ .017. (PDF) [file pone.0214069.s004.pdf]

Table 4. Sense of Agency and Classic Narcissistic Self.

**Subscale**	M_{high}	***SD***	M_{low}	***SD***	***t*-test**	***p***
**Baseline action**	-59.35	102.20	-61.26	94.64	-.097	.462
**Agency action**	-82.10	108.20	-102.44	119.57	-.883	.190
**Baseline outcome**	-65.50	65.68	-60.20	58.04	.430	.334
**Agency outcome**	-19.62	118.50	-4.00	142.45	.589	.279
**Total binding**	68.63	106.22	91.46	147.00	.867	.194

One-tailed *t*-tests for intentional binding conditions, first (high, $N = 44$) versus fourth (low, $N = 56$) percentiles in the narcissism subscale scores for classic narcissistic self. **Significance** $p \leq .017$.
