# Supplementary material for: Dopamine and sense of agency: Determinants in personality and substance use
Source: PLoS One. 2019 Mar 19;14(3):e0214069. doi: 10.1371/journal.pone.0214069 (PMC6424396; doi:10.1371/journal.pone.0214069)
Supplement: S5 Table — Absolute number of users. (PDF) [file pone.0214069.s005.pdf]

Table 5. Consumed Substances (Ever).

	**Cannabis**	**Ecstasy**	**Amphetamine**	**Mushrooms**	**LSD**	**Cocaine**	**Ketamine**	**> Cannabis**
yes	131	63	57	44	43	49	34	81
No	78	146	152	165	166	160	175	129

Absolute number of users.
