# Supplementary material for: Dopamine and sense of agency: Determinants in personality and substance use
Source: PLoS One. 2019 Mar 19;14(3):e0214069. doi: 10.1371/journal.pone.0214069 (PMC6424396; doi:10.1371/journal.pone.0214069)
Supplement: S6 Table — One-tailed t-tests for sense of agency in users versus controls for each substance. Significance p ≤ .017. Drug users = consumption beyond cannabis. (PDF) [file pone.0214069.s006.pdf]

Table 6. Differences in Sense of Agency (Overall Binding) between Drug Users and Controls.

**Substance**	M_{users}	SD	$M_{control}$	SD	***t*-test**	p
**Cannabis**	67.51	136.31	111.29	133.13	2.227	**.014**
**Ecstasy**	51.87	139.51	97.77	133.28	2.203	**.015**
**Amphetamine**	60.80	141.07	92.84	134.12	1.489	.069
**Mushrooms**	67.49	132.90	88.58	137.47	.898	.185
**LSD**	66.29	147.33	88.63	133.63	.935	.176
**Cocaine**	43.74	124.84	96.38	137.85	2.340	**.010**
**Ketamine**	76.42	138.23	85.63	136.45	.359	.360
**> Cannabis**	55.37	134.50	103.15	134.26	2.454	**.008**

One-tailed t-tests for sense of agency in users versus controls for each substance. **Significance** $p \leq .017$.

Drug users = consumption beyond cannabis.
